# Authoritative subspecies diagnosis tool for European honey bees based on ancestry informative SNPs

**DOI:** 10.1186/s12864-021-07379-7

**Published:** 2021-02-03

**Authors:** Jamal Momeni, Melanie Parejo, Rasmus O. Nielsen, Jorge Langa, Iratxe Montes, Laetitia Papoutsis, Leila Farajzadeh, Christian Bendixen, Eliza Căuia, Jean-Daniel Charrière, Mary F. Coffey, Cecilia Costa, Raffaele Dall’Olio, Pilar De la Rúa, M. Maja Drazic, Janja Filipi, Thomas Galea, Miroljub Golubovski, Ales Gregorc, Karina Grigoryan, Fani Hatjina, Rustem Ilyasov, Evgeniya Ivanova, Irakli Janashia, Irfan Kandemir, Aikaterini Karatasou, Meral Kekecoglu, Nikola Kezic, Enikö Sz. Matray, David Mifsud, Rudolf Moosbeckhofer, Alexei G. Nikolenko, Alexandros Papachristoforou, Plamen Petrov, M. Alice Pinto, Aleksandr V. Poskryakov, Aglyam Y. Sharipov, Adrian Siceanu, M. Ihsan Soysal, Aleksandar Uzunov, Marion Zammit-Mangion, Rikke Vingborg, Maria Bouga, Per Kryger, Marina D. Meixner, Andone Estonba

**Affiliations:** 1 Eurofins Genomics Europe Genotyping A/S (EFEG), (Former GenoSkan A/S), Aarhus, Denmark; 2 Laboratory Genetics, University of the Basque Country (UPV/EHU), Leioa, Bilbao Spain; 3 Swiss Bee Research Center, Agroscope, Bern, Switzerland; 4 Laboratory of Agricultural Zoology and Entomology, Agricultural University of Athens, Athens, Greece; 5 Department of Molecular Biology and Genetics, Aarhus University, Aarhus, Denmark; 6 Institutul de Cercetare Dezvoltare pentru Apicultura SA, Bucharest, Romania; 7 University of Limerick, Limerick, Ireland; 8 CREA Research Centre for Agriculture and Environment, Bologna, Italy; 9 BeeSources, Bologna, Italy; 10 Veterinary Faculty, University of Murcia, Murcia, Spain; 11 Croatian Ministry of Agriculture, Zagreb, Croatia; 12 Department of Ecology, Agronomy and Aquaculture, University of Zadar, Zadar, Croatia; 13 Breeds of Origin, Haz-Zebbug, Malta; 14 MacBee Association, Skopje, North Macedonia; 15 Faculty of Agriculture and Life Sciences, University of Maribor, Maribor, Slovenia; 16 Yerevan State University, Yerevan, Armenia; 17 Department of Apiculture, Agricultural Organization ‘DEMETER’, Thessaloniki, Greece; 18 Division of Life Sciences, Major of Biological Sciences, and Convergence Research Center for Insect Vectors, Incheon National University, Incheon, Korea; 19 Institute of Biochemistry and Genetics, Ufa Federal Research Centre of the Russian Academy of Sciences, Ufa, Russia; 20 University of Plovdiv “Paisii Hilendarski”, Plovdiv, Bulgaria; 21 Agricultural University of Georgia, Tbilisi, Georgia; 22 Ankara University, Ankara, Turkey; 23 Federation of Greek Beekeepers’ Associations, Larissa, Greece; 24 Düzce University, Düzce, Turkey; 25 University of Zagreb, Zagreb, Croatia; 26 Hungarian Bee Breeders Association, Budapest, Hungary; 27 Division of Rural Sciences and Food Systems, Institute of Earth Systems, University of Malta, Msida, Malta; 28 Österreichische Agentur für Gesundheit und Ernährungssicherheit GmbH, Wien, Austria; 29 Cyprus University of Technology, Limassol, Cyprus; 30 Agricultural University of Plovdiv, Plovdiv, Bulgaria; 31 Centro de Investigação de Montanha (CIMO), Instituto Politécnico de Bragança, Bragança, Portugal; 32 Shulgan-Tash Nature Reserve, Burzyansky District, Russia; 33 Tekirdag University, Tekirdag, Turkey; 34 Landesbetrieb Landwirtschaft Hessen, Bee Institute Kirchhain, Kirchhain, Germany; 35 Faculty of Agricultural Sciences and Food, University Ss. Cyril and Methodius, Skopje, Republic of Macedonia; 36 Department of Physiology and Biochemistry, University of Malta, Msida, Malta; 37 Department of Agroecology, Aarhus University, Slagelse, Denmark

**Keywords:** *Apis mellifera*, European subspecies, Conservation, Machine learning, Prediction, Biodiversity

## Abstract

**Background:**

With numerous endemic subspecies representing four of its five evolutionary lineages, Europe holds a large fraction of *Apis mellifera* genetic diversity. This diversity and the natural distribution range have been altered by anthropogenic factors. The conservation of this natural heritage relies on the availability of accurate tools for subspecies diagnosis. Based on pool-sequence data from 2145 worker bees representing 22 populations sampled across Europe, we employed two highly discriminative approaches (PCA and F_ST_) to select the most informative SNPs for ancestry inference.

**Results:**

Using a supervised machine learning (ML) approach and a set of 3896 genotyped individuals, we could show that the 4094 selected single nucleotide polymorphisms (SNPs) provide an accurate prediction of ancestry inference in European honey bees. The best ML model was Linear Support Vector Classifier (Linear SVC) which correctly assigned most individuals to one of the 14 subspecies or different genetic origins with a mean accuracy of 96.2% ± 0.8 SD. A total of 3.8% of test individuals were misclassified, most probably due to limited differentiation between the subspecies caused by close geographical proximity, or human interference of genetic integrity of reference subspecies, or a combination thereof.

**Conclusions:**

The diagnostic tool presented here will contribute to a sustainable conservation and support breeding activities in order to preserve the genetic heritage of European honey bees.

**Supplementary Information:**

The online version contains supplementary material available at 10.1186/s12864-021-07379-7.

## Background

Honey bees (*Apis mellifera* L.) are the most important managed pollinators and currently under threat due to a multitude of pressures worldwide [1, 2]. The species shows considerable variation across its natural range and is comprised of at least 30 described subspecies belonging to different evolutionary lineages [3–6]. Europe holds a large fraction of this honey bee diversity with numerous endemic subspecies representing four evolutionary lineages, namely the African lineage (A), Central and Eastern European lineage (C), Western and Northern European lineage (M), and Near East and Central Asian lineage (O) [7, 8]. However, this diversity and the natural distribution range of European honey bees have been influenced by anthropogenic factors to an extent that several locally adapted populations are at risk due to introgression and crossbreeding [9–11]. Large-scale queen breeding, commercial trade and long distance migratory beekeeping may reduce genetic diversity and can lead to genetic homogenization of admixed populations [9, 12] and potential subsequent loss of local adaptations. In fact, it has been demonstrated that locally adapted honey bees have higher survivability [13] from which follows that the conservation of the underlying genotypic variation must be a priority for the long-term sustainability of populations [14]. To conserve the honey bees’ natural heritage and thereby its adaptive potential to future global change, there is a need to promote the sustainable breeding of certified local subspecies.

Numerous conservation efforts for native honey bees have been initiated across Europe [9, 10, 15, 16]. The success of such conservation efforts including genetic improvement programs [17, 18] depends on mating within the population of interest, which is complicated by the honey bees’ mating system where virgin queens mate freely with multiple drones from surrounding colonies [19, 20]. Beyond the use of isolated mating apiaries or artificial insemination, successful mating control measures can include different management techniques of queens and drones [21] and regular monitoring of genetic origin and parentage. In some countries and regions in Europe, queen importations are restricted to the native honey bee subspecies [22, 23] or ecotypes [24, 25]. In such instances, when trading queens or colonies across national borders, queen origin needs to be verified. Additionally, authentication of the genetic origin of bee products in terms of a certifiable native bee label, could help beekeepers to better market their hive products [26]. Thus, to implement effective border control, increase economic value of bee products and to support informed conservation and breeding management decisions across Europe, there is a demand for diagnostic genetic test to reliably infer the subspecies of origin.

With the advances of high-throughput sequencing and genotyping technology in the last decade, reference genomes, whole-genome sequence data, and thousands of individual genotypes are now available for many species. Within these oftentimes massive data sets, it is possible to mine for highly informative single nucleotide polymorphisms (SNPs) that can then be exploited to genotype a larger number of individuals [27, 28]. Such genotyping panels based on a selected set of informative SNPs have been developed for numerous species, including humans, and can be used to infer introgression, genetic ancestry, population structure, genetic stock identification, and food forensics [29–31].

Different approaches have been used to select informative SNPs from larger genotyping panels or sequence data (reviewed in [32, 33]). The most common and popular method for selection is population differentiation as estimated by F_ST_, which is based on allele frequency differences between populations expressing the variation among populations relative to the total population [34, 35]. Principal Component Analysis (PCA) has also been employed to identify informative SNPs, since it reduces feature dimensionality while only losing little information and is particularly advantageous with complex population structures [28, 36]. Given a set of informative SNP markers, supervised classification and so-called assignment tests are employed whereby an individual is assigned to predefined classes (i.e., subspecies or populations of origin). Classical applications of assignment testing in population genetics first used supervised parametric likelihood-based approaches [37, 38]. Recently, new methods, together referred to as supervised machine learning (ML), have emerged in computational population genomics [39]. The general approach for any supervised ML classifiers is to split the data into a reference (training) set to ‘learn’ a function that can discriminate between the given data classes [40]. This function is then used to predict the probability of an ‘unknown sample’ (test) of belonging to any given class (e.g. subspecies). The accuracy of the classification, expressed as the proportion of test individuals correctly classified to their population of origin, is influenced by the properties of the training data set (i.e., number of samples, genetic diversity, levels of population differentiation, degree of overlap in data distribution and quality of reference samples) [41]. ML classifiers aim to optimize the predictive accuracy of an algorithm rather than performing parameter estimation of a probabilistic model, and they have the potential to be agnostic to the assessment of the given dataset, i.e. without assumptions of the processes leading to differentiation, including the evolutionary history [39].

For honey bees, different SNP panels have been designed, for instance to identify and estimate C-lineage introgression in M-lineage subspecies *A. m. iberiensis* and *A. m. mellifera* [15, 42–46]. The latter subspecies is native to northern and western Europe and once occupied a large fraction of the European territory, but is now threatened and even has been completely replaced in much of its range [10, 47, 48]. Moreover, SNP panels have also been developed to infer the level of Africanization and ancestry in honey bees of the New World and Australia [46, 49, 50]. However, for most *A. mellifera* subspecies, whose populations have been genetically examined to a lesser extent or not at all, molecular knowledge at this level of detail is still lacking. These subspecies and locally adapted populations or ecotypes appear more vulnerable due to the extant multiple threats to honey bees.

The SmartBees project was initiated with the purpose of developing new tools to describe and conserve honey bee diversity in Europe. We have designed a molecular tool consisting of highly informative SNP markers suitable for assigning honey bee individuals to their subspecies of origin, based on a comprehensive sampling of European honey bee diversity. Based on pool-sequence data from 1995 worker bees representing 22 populations, four evolutionary lineages and 14 subspecies, we selected 4400 informative SNPs employing two powerful and commonly used approaches (F_ST_ and PCA). Of these, 4165 SNPs, for which probes could be designed and which passed the BeadChip decoding quality metric, were genotyped in 3903 individual bees using the Illumina Infinium platform. Final quality control filtering left 4094 reliable SNPs to build a statistical model using machine learning (ML) algorithms for assignment of European honey bees to 14 different genetic origins. The best model was the Linear Support Vector Classifier (Linear SVC) which could correctly assign 96.2% of the tested samples to their genetic origin. Thus, the here presented method accurately identifies European subspecies, which is crucial to support management strategies in sustainable honey bee breeding and conservation programs.

## Results

### Samples and pool-sequencing

A total of 22 populations representing the four European evolutionary lineages and 14 subspecies have been sampled from their native ranges throughout Europe and adjacent regions (Tables 1 and S1). Each selected population included up to 100 worker bees from unrelated colonies, totaling 2145 samples, which represents the most comprehensive sampling effort for the study of European honey bees to date. The samples from each population were homogenized, pooled and their DNA extracted. Sequencing on an Illumina HiSeq 2500, produced 1.6 billion paired-end fragments (3.2 billion individual reads) with an average read length of 125 bp, and a total genome depth of coverage of 2800x. Sequencing and variant statistics can be found in Table S2.

**Table 1:** Samples individually genotyped for subspecies classification (N_TOT_ = 3896) consisting of individual samples from the pool sequencing (in bold, *N* = 1998, excluding 62 outliers) and new independent samples (*N* = 1908). Samples were collected from their native range and labelled based on previous studies, morphometric analysis or local knowledge (see Methods sections and Table S1). 70% of pool sequencing samples (*N* = 1391) were used as training data for building the model, while the remaining 30% (*N* = 597) together with the independent samples (N_Total_ = 2505) were considered as out-of-sample data for subsequent validation

Evolutionary lineage	Subspecies	Sampling country	Pool name / Sampling group	***N***	***N***_**TOT**_
A	*A. m. ruttneri*	Malta	**rut_mlt**	91	187
			MLT	96	
C	*A. m. adami*	Crete, Greece	**ada_grc**	82	82
	*A. m. carnica*	Austria & Hungary	**car_aut_hun**	93	825
		Croatia & Slovenia	**car_svn_hrv**	95	
		Croatia	HRV	94	
		Denmark	DNK	89	
		France	FRA	8	
		Germany	GER	282	
		Poland	POL	40	
		Serbia	SRB	49	
		Slovenia	SVN	75	
	*“A. m. carpatica”*	Romania & Moldova	**carp_rou_mda**	86	86
	*A. m. cecropia*	France	FRA	4	4
		Greece	**cec_grc**	93	140
			GRC	47	
	*A. m. ligustica*	Italy	**lig_ita**	84	143
			ITA	59	
	*A. m. macedonica*	N. Macedonia & N-Greece	**mac_mkd_grc**	86	429
		Greece	GRC	49	
		N. Macedonia	MKD	96	
		Germany	GER	198	
	*“A. m. rodopica”*	Bulgaria	**rod_bgr**	84	84
M	*A. m. iberiensis*	Spain & Portugal	**ibe_esp_west_prt**	94	460
		Spain	**ibe_esp_eus**	96	
			**ibe_esp_north**	91	
			**ibe_esp_south**	64	
			ESP	115	
	*A. m. mellifera*	Belgium	BEL	96	1066
		Denmark	**mel_dnk**	96	
			DNK	97	
		Finland	FIN	15	
		France	FRA	49	
		Ireland	**mel_irl**	96	
		Isle of Man	**mel_imn**	92	
		Norway	NOR	12	
		Poland	POL	33	
		Russia	**mel_rus**	96	
		Scotland	SCT	280	
		Sweden	SWE	8	
		Switzerland	**mel_che**	96	
O	*A. m. anatoliaca*	Turkey	**ana_tur**	94	94
	*A. m. remipes*	Armenia	**rem_arm**	90	90
	*A. m. caucasia*	Poland	**cau_tur_geo**	96	113
		Denmark	DNK	4	
		NE-Turkey & Georgia	POL	13	
	*A. m. cypria*	Cyprus	**cyp_cyp**	93	93
**Total**					**3896**

### Selected SNPs

While main evolutionary lineages were easily differentiated with only few SNPs (Figure S1A), it was more challenging to differentiate closely related subspecies with a reduced number of genetic markers. Given the complex, hierarchical population structure of European honey bees, we employed two powerful and commonly used approaches, PCA (Figure S1) and F_ST_, to identify the most discriminant markers to differentiate subspecies of European honey bees (see details in Methods and supplementary materials and methods). Based on the variants infered from the pool-sequence data, we selected 4400 informative SNPs, of these, a total of 4165 SNPs passed the decoding quality metric for genotyping using the Illumina Infinium custom-designed BeadChip, indicating that 99% of the originally submitted probes were suitable for genotyping. The SNPs are distributed across all of the 16 honey bee chromosomes as well as in unplaced contigs (Table S3), with an average distance between SNPs of 64 kb. SNP information and genomic position of the 4165 SNPs selected to differentiate European honey bee subspecies are presented in Additional file 1.

### Sample genotyping and visualization

Of the 4165 SNPs, 4094 were successfully genotyped in 3896 individual bees using Illumina Infinium BeadChip technology (Table 1). With only 71 SNPs never producing any data, the genotyping success rate (SNP validation) rate was 98%. The average call rate per individual was 0.87, varying among samples of every subspecies from 0.84 in *A. m. cypria* to 0.89 *in A. m. adami* (Table S4). More than one-third of the samples have a call rate exceeding 0.9.

**Fig. 1:**
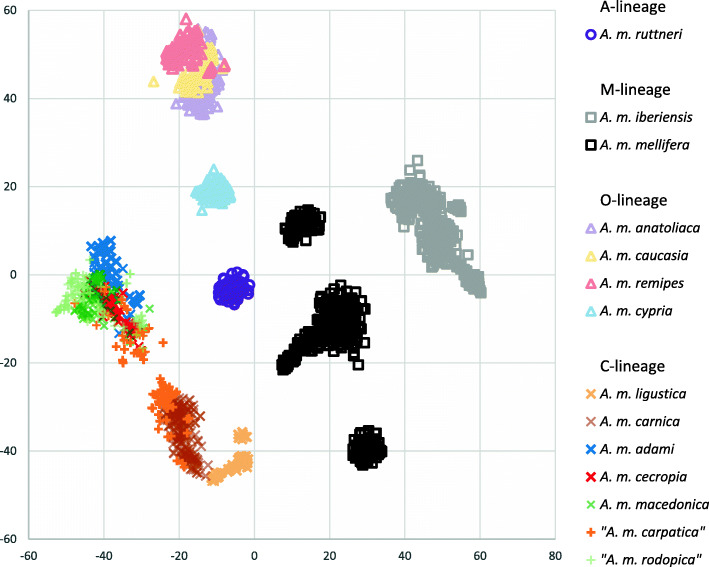
Visualization using a t-SNE manifold plot of the 1988 honey bee samples from the pool sequencing individually genotyped for 4094 SNPs. Samples have been color-coded according to the subspecies reference populations corresponding to the 14 classes used for subsequent supervised machine learning classification

The genotype data of the individuals from the pool sequencing is visualized in a t-SNE plot [51] that reduces high-dimensional data to a two-dimensional map where each individual is represented by a point (Fig. 1). The genotyped samples were grouped in several separated clusters according to their evolutionary lineage or subspecies of origin (Fig. 1). Within each lineage, most of the individuals from the same geographic origin were closely grouped together and generally well separated from neighboring groups. The only A-lineage subspecies in our study, *A. m. ruttneri*, was placed in the center intermediate to the other clusters. In the O-lineage, *A. m. cypria* bees were well separated from *A. m. anatoliaca, A. m. caucasia* and *A. m. remipes*, which appear less well differentiated. The two subspecies of the M-lineage were well differentiated, with *A. m. mellifera* populations grouped in three subclusters separating the distant (Burzyan region, Russia, top *A. m. mellifera* cluster in Fig. 1) or isolated (Læsø island, Denmark, bottom *A. m. mellifera*) sampling regions. C-lineage samples grouped into three subclusters: (i) *A. m. ligustica*, (ii) *A. m. carnica* bees including part of the “*A. m. carpatica”* samples and (iii) a heterogeneous subcluster of *A. m. macedonica*, *A. m. cecropia*, *A. m. adami*, “*A. m. rodopica*” and the rest of “*A. m. carpatica*” bees. A t-SNE plot with sample labels according to their pool of origin is presented in Figure S2.

### Sample classification using machine learning

We employed machine learning (ML) methods to build a model for the classification and assignment of European honey bees to its subspecies of origin. Out of the tested ML algorithms, the best performing model was the Linear SVC (Table S5). The model calculates the prediction probability for a sample to belong to any of the 14 reference populations. Each test sample was classified into the subspecies which showed the highest prediction probability ranging from as low as 0.29 to 1.0 with a median of 0.98 (Figure S3).

**Fig. 2:**
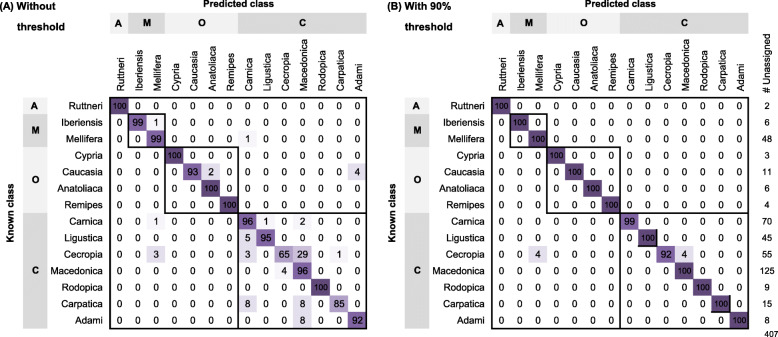
Confusion matrix for test samples (out-of-sample data, *N* = 2505) showing the (rounded) percentages of truly assigned individuals (diagonal) and percentages of individuals assigned to a different subspecies (misclassified; upper and lower triangles). **a** Assignment based on the highest prediction probability classifies each of the test individuals to a subspecies, while **b** using a probability threshold of 90% some samples are considered “unassigned” and excluded from the confusion matrix

A confusion matrix was used to summarize, describe and visualize the performance of the Linear SVC classification model on a set of test data (out-of-sample data, *N* = 2505) for which the true values (subspecies) were known. For the lineages, the model is capable of predicting all samples with 100% accuracy (Figure S4). For the subspecies, the confusion matrix revealed that for most of them the model accurately predicted the ancestry of the test samples (N = 2505), with only a few exceptions (Fig. 2a). The accuracy ranged from 65 to 100%, indicating that some subspecies are easier to distinguish than others. In total 96.2% of test samples were correctly predicted, while 95 individuals (3.8%) were misclassified, i.e.*,* predicted by the model with a different subspecies than the labeled one (true values), for instance: four *A. m. ligustica* bees were predicted as *A. m. carnica,* two “*A. m. carpatica*” bees each as either *A. m. carnica* or *A. m. macedonica,* and 23 *A. m. cecropia* bees were predicted as *A. m. macedonica*.

The model predicts the probability that a given sample belongs to one of the 14 subspecies under study. On this basis, the test samples were assigned to a certain subspecies based on the highest prediction probability, even if the probability was low (see above). Therefore, with the purpose of increasing the certainty of classification we set a probability threshold, so to ensure that only samples very likely belonging to any of the 14 subspecies were assigned, while test samples with low prediction probabilities were considered unassigned. In Fig. 2b, we show an example of setting a probability threshold at 90%. By setting this threshold, we increased the proportion of truly assigned samples from 96.1 to 99.6%, while the misclassification rate fell from 3.9 to 0.4%. However, 407 of the test individuals remained “unassigned”, for instance, 22 out of the 23 *A. m. cecropia* bees predicted as *A. m. macedonica* were no longer considered misclassified but enter the unassigned category.

## Discussion

In this study, we performed a large-scale and comprehensive sampling following a standardized procedure, and aimed to capture as much of the honey bee genetic diversity in Europe as possible by deep-sequencing of pooled populations. Further, we applied two powerful SNP selection methods [32, 33] to address diversity at different levels of differentiation (lineages, subspecies, populations). Subsequently, these ancestry informative markers were employed to build a model to classify samples of European honey bees into subspecies.

The considerable honey bee diversity poses a challenge when it comes to providing a discriminative tool applicable across Europe. The four European lineages were easily distinguished genetically with only 200 SNPs due to their ancient divergence [52], but difficulties arose at a lower hierarchical level of differentiation. Subspecies from the same evolutionary lineage diverged only recently [53] and are, thus, genetically very close. Moreover, there are some areas in Europe where *A. mellifera* subspecies variation has not yet been exhaustively described, while in others human-mediated introgression contributes to blurring the natural boundaries between subspecies [42, 48, 54]. National breeding programs can also disrupt the natural gene flow and may contribute to changing the genetic background of the original subspecies [11, 12, 55, 56]. In fact, in our study applying a stringent filtering option we only identified few unique SNPs that were exclusive to one population. Similarly, other population genomics studies have found a high degree of allele sharing across and within evolutionary lineages [7, 53]. In contrast, we found variation in the average call rate per individual between subspecies which may, in part, be explained by the presence of null alleles (alleles producing no signal), suggesting sequence variation or subspecies-specific deletions within the probe site. Probes that did not work for certain subspecies (i.e. missing data), in fact, contain valuable information and even enriched our model.

We employed a machine learning (ML) approach to build a model for subspecies classification. ML takes advantage of high dimensional input and provides an improvement of prediction accuracy in a model-free approach [39, 40]. In this way, subtle differences can be revealed which was particularly relevant in our study, due to the high number of closely related subspecies we wanted to discriminate. Our best performing model was Linear SVC, member of the family of Support Vector Machines (SVMs), which are known to generalize well because they are designed to maximize the margin between any two classes (subspecies) [57]. Typical biological applications of SVMs include protein function prediction, transcription initiation site prediction and gene expression data classification (reviewed in 57). In the field of population genetics, a thorough ML approach to select the best model is generally not yet commonly implemented, although specific models have been developed for ancestry inference [58, 59]. Here, we employ a comprehensive ML approach based on genotype data for honey bee subspecies diagnosis.

Despite the comprehensive sampling effort, the careful SNP selection and the application of the latest classification methods, some limits remain in the diagnostic system. For instance, within the C-lineage we have experienced problems in differentiating samples according to the alleged subspecies. Such misclassification of individuals can be explained by various factors coming together: (i) this lineage is of comparatively recent origin [53] and (ii) consists of multiple highly interrelated subspecies within close geographical proximity (see Figure S1D); (iii) the taxonomic status of some populations has not yet been fully resolved [60–62]; and (iv) the genetic background of some populations is being altered by introgression due to human interference [63]. Furthermore, labelling errors of the out-of-data samples could not be ruled out as an additional source of misclassification, especially if we refer to those samples for which the model predicted a different subspecies with high probability. Supervised ML relies on the qualities of the reference data for classification, thus, in the future, we aim to refine the training data to improve the model prediction accuracy and reduce the misclassification rate.

It is also important to note, that by setting a probability threshold for the assignment of any subspecies, the misclassification rate was reduced, for some subspecies considerably. While such a threshold increases the confidence in subspecies prediction, it also implied, however, that quite a few individuals were left “unassigned”. What threshold is used as a cut-off for subspecies classification depends on the specific circumstances and the application. For example, for the conservation of a small endangered population the threshold might be set lower in order to maintain genetic diversity, than for instance in a pure breeding line under selection for specific traits.

Overall, earlier methods based on morphometry, mtDNA variation, microsatellite loci, or even SNPs have been effective in differentiating between evolutionary lineages and, to some extent, between subspecies of the same lineage [22, 42, 45, 64–67]. Yet, our diagnostic tool is the most comprehensive tool to date to reliably classify European honey bees into subspecies in a single analysis. Moreover, the advantage of our approach is that it is a dynamic tool that can be updated to include more subspecies by genotyping new samples and adding their data to rebuild a classification model using ML with additional subspecies. Ongoing research indicates that this approach is applicable to *A. m. siciliana* from Sicily. Furthermore, individual bees from South Africa tested with our system were rejected as being of European origin (*i. e.*, low prediction probability to any of the subspecies). This dynamic tool, therefore, could easily incorporate new populations to be discriminated, and would even have the potential to be optimized to differentiate populations/ecotypes within subspecies, or to evaluate the degree of introgression.

## Conclusions

The main finding of the study is that our model can classify bees into each of the European subspecies with high accuracy. Consequently, as the bees included in this project were collected in a vast area ranging from Russia and Armenia in the East to Portugal in the West, and from Malta in the South to Scotland in the North, we conclude that much of the natural diversity of European honey bees can still be considered extant, in spite of human interference since more than 150 years. The in situ conservation of this genetic heritage is our duty [68], and we believe that the honey bee subspecies diagnostic tool presented will make a useful contribution. It is of value in an array of applications: for beekeepers who want to know the subspecies of their bees; for conservation managers in Europe, where subspecies diagnosis is essential to monitor the hybridization rate of colonies within conservatories; for veterinarians to control queen trade; for bee breeders to certify the subspecies origin of their queens; and for beekeepers to authenticate their bee products.

## Methods

### Pool-sequencing samples

For this study, in total 22 populations have been sampled, all within their native range (Tables 1 and S1), and are referred to as different subspecies and genetic origins according to the classification of Ruttner [8] and the most recent revision of the genus *Apis* by Engel [62]: *A. m. iberiensis* Engel 1999*, A. m. mellifera* Linnaeus 1758*, A. m. carnica* Pollman 1879*, A. m. caucasia* Pollmann 1889*, A. m. ligustica* Spinola 1806*, A. m. macedonica* Ruttner 1988*, A. m. cecropia* Kiesenwetter 1860*, A. m. cypria* Pollman 1879*, A. m. adami* Ruttner 1975*, A. m. anatoliaca* Maa 1953*, A. m. remipes* Gerstaecker 1862; in addition we include *A. m. ruttneri* Sheppard et al. 1997 [69], “*A. m. carpatica”* Foti 1965 [60], and “*A. m. rodopica*” Petrov 1991 [61]. There exist some uncertainty and unresolved taxonomic status of some populations, and subspecies descriptions in literature have not always been performed according to the standards laid down in the International Code of Zoological Nomenclature (ICZN) [62]. Thus, different views are found in literature to what is to be considered a subspecies or ecotype. In this paper, we do not aim to resolve or justify any classification. Finally, we considered 14 subspecies/genetic origins (listed above) for our diagnostic tool, which were used as categories in the machine learning classification model.

Each selected population included up to about 100 (ranging from 86 to 100) worker bees from unrelated colonies that were used for subsequent pool-sequencing. Effort was undertaken to cover the entire distribution range of any subspecies, while taking into account within-subspecies variability when appropriate. We focused on collecting representative samples for each subspecies by primarily sampling from beekeepers that were known not to import bees in order to minimize the risk of including hybrids. Moreover, we only chose one worker bee per apiary to avoid related individuals and to include as much diversity per population as possible. Also in order to secure the subspecies-origin of the collected samples, in some cases (where possible), a morphometric analysis was performed and/or we relied on already genotyped bees [55, 65, 66, 70–72]. Detailed information on sample origin and respective references are presented in Table S1.

### DNA extraction, library preparation, and pool-sequencing

The heads or thoraxes of up to 100 bees (Table S1) from each pool were homogenized, DNA was extracted from all samples by using a magnetic bead-based purification method (NucleoMag® Blood 100 μL, Macherey-Nagel, Germany). Subsequently, sequencing libraries of each pool-DNA were constructed with the TruSeq DNA PCR-Free library preparation kit and sequenced on an Illumina HiSeq 2500 platform. Bioinformatic processing, including trimming, mapping and variant calling of the generated pool sequence data, was performed using best practices and standard software (details in supplementary material and methods). The pipeline for the analysis of the pool sequence data is available at https://github.com/jlanga/smsk_popoolation.

### Selection of ancestry informative markers

Several studies have selected a limited number of SNPs to differentiate between the main evolutionary lineages [15, 45, 46], however, for closely related subspecies more markers and a more refined selection approach are needed. Thus, we used two different approaches (PCA and F_ST_) [28, 34] to identify and select informative SNPs, in order to capture the most discriminant markers at different levels: (i) SNPs to differentiate the four main evolutionary lineages, (ii) SNPs to discriminate subspecies within evolutionary lineages, and (iii) SNPs to identify specific populations within subspecies (e.g. ecotypes).

First, we created a matrix with the minor allele frequencies for each SNP and sequenced pool, which was used to perform PCA to select SNPs that differentiate the main evolutionary lineages (Figure S1A). Second, PCA was performed separately on the subsets of pools from each lineage in order to select informative SNPs to discriminate subspecies within each lineage (Figure S1B-D). We used the FactoMineR R package [73] and custom-made R scripts to select at each hierarchical level the SNPs with the highest contributions to the significant PCs. Using this procedure, 300 PCA-informative SNPs were selected for discriminating the four evolutionary lineages, 200 SNPs for the M-lineage, 600 SNPs for the O-lineage and 1100 SNPs for the most complex C-lineage (Figure S1D). Preliminary simulations using allele frequencies from the pool-sequencing revealed that this approach was highly effective in discriminating lineages and subspecies (Figure S1).

To select additional SNPs that can differentiate between pools, pairwise F_ST_ values [74] between all population were calculated for each SNP with two settings (loose and stringent options) using PoPoolation2 [75]. The loose setting option will return more SNPs with less certainty and lower quality, which in turn potentially reduces genotyping success. This drawback is counterbalanced, since the loose option increases the chance of identifying highly informative population-specific (unique) SNPs. For either setting option (loose and stringent), the pairwise F_ST_ values of each pool against all other pools were summed up for each SNP and then ranked according to the highest summed F_ST_ value. A fixed and unique SNP in one pool is expected to have a maximum sum of 21, which means this variant is only present in this specific population. A reasonable trade-off between unique and reliable SNPs was achieved by selecting the top 20 SNPs with the highest summed F_ST_ from the loose option and the top 80 SNPs from the stringent option for each pool. With 22 pools, a total of 2200 informative population-specific SNPs were selected using F_ST_.

Overall, 4400 ancestry informative SNPs were selected based on PCA and F_ST_ (Table S3). These highly informative markers are not only important for the assignment of individuals to subspecies as presented in this study, but, because of their varied allele frequencies in different populations, they can be used, for instance, for classification of new subspecies and for further follow-up studies.

### Probe design

Probes for the 4400 selected SNPs were evaluated for genotyping on the Illumina Infinium platform using Illumina’s DesignStudio® software which requires as input the flanking region of 50 bp up and downstream of each SNP. SNPs were discarded if no probe could be designed in the flanking region or if the probes had more than one hit when aligned to the honey bee reference genome. The final list of 4197 SNPs was submitted to Illumina for probe design and production. The SNPs are distributed across all of the 16 honey bee chromosomes as well as in unplaced contigs (Table S3; Additional file 1), with an average distance between SNPs of 64 kb.

### Validation samples and genotyping

A total of 3958 individual bees were genotyped for the selected SNPs, including 2050 same individual worker bees that were used for pool sequencing, as well as 1908 newly collected individuals (Table 1). These new additional samples were received from several different sources and of variable quality, including whole honey bees in ethanol, honey bees squeezed on FTA cards, tissue samples from flight muscle or purified DNA. These originated from SmartBees breeding apiaries [76] and from colonies examined for Varroa-sensitive hygienic behavior within the SmartBees project [77]. The samples were genotyped using the custom-made BeadChip array Infinium iSelect XT 96. The results were analyzed using Illumina’s GenomeStudio® software, and the genotypes of each sample were exported for further analysis. For an initial visualization of the genotyping results, we created t-distributed stochastic neighbor embedding (t-SNE) manifold plots. This technique visualizes high-dimensional data by giving each data point a location in a two- or three-dimensional map [78]. Outliers and samples that were labeled as one subspecies, but were clearly grouped with another cluster, were removed, in total 62 samples, leaving *N* = 1988 pool sequence reference samples. This was done with the objective to create a high-quality and representative reference data set for subspecies assignment.

### Sample classification using machine learning (ML) algorithms

In order to build a model to classify and predict the subspecies assignment of unknown samples of European honey bees, we employed ML methods using the scikit-learn python environment [79]. First, the 1988 genotyped individuals from the pools were shuffled, then 70% of them (*N* = 1391) were used as training data. The remaining 30% (*N* = 597), together with the additional newly collected individuals (*N* = 1908) were considered as out-of-sample data (N_Total_ = 2505) for subsequent validation (Table 1) [40]. Different supervised ML algorithms were tested, including RandomForest, LogisticRegression, SupportVector Machine (SVM), and Linear SupportVectorClassifier (SVC) (Table S5; detailed information on model selection in supplementary materials and methods). Briefly, the genotype data was converted to a matrix compatible with machine learning (one-hot encoding) [80]. Class information such as lineage and subspecies of each sample was added to the matrix, which was used to train the different machine learning models to predict the sample ancestry. Linear SVC was one of the best performing models according to average accuracy estimated using cross-validation and was finally selected (Table S5, Figure S5).

After training the Linear SVC model, it was used to classify out-of-sample data (*N* = 2505). Samples were classified according to the highest prediction probability belonging to any of the subspecies. A confusion matrix [81] was created to summarize and visualize the performance on out-of-sample data for which the true values are known. Each row of the matrix represents the true class, while each column represents the predicted class based on the highest probability for each subspecies. The resulting percentages compare a list of expected values with a list of predictions from the model.

In order for the model to be applied in practical conservation and breeding, we defined a threshold of 90% based on the observed distribution of the prediction probabilities (Figure S3), which are in accordance with values found in bee literature [43, 82]. If the prediction probability for any given sample is less than the threshold of 90%, it is considered “unassigned”, while if it exceeded the threshold it was assigned to the respective subspecies.

## Supplementary Information

**Additional file 1.** Honey bee subspecies informative markers. SNP information and genomic position of the 4165 SNPs selected in this study to differentiate European honey bee subspecies.

**Additional file 2: Supplementary materials and methods.** Supplementary materials and methods describing in detail the datasets used, the laboratory methods, the bioinformatic pipeline, the SNP selection approach, and the sample classification using Machine Learning algorithms.

**Additional file 3: Figure S1.** PCA-Plots with the PCA-selected SNPs and 100 simulated individuals based on allele frequencies of the pools. **(A)** Using 300 SNPs, the evolutionary lineages M, C and O were well separated with the first two PCs, while lineage A can be differentiated with the third component (not shown). **(B)** Within the M-lineage, harboring only two European subspecies (*A. m. mellifera* and *A. m. iberiensis*), the first PC using 200 SNPs already contributes sufficient information. **(C)** In the O-lineage, four subspecies are represented that are separated with 600 SNPs using 4 PCs. **(D)** For lineage C that contains numerous subspecies (subspecies complex), 1100 SNPs were selected to obtain a better resolution.

**Additional file 4: Figure S2.** Visualization using a t-SNE manifold plot of the 1988 honey bee samples from the pool sequencing individually genotyped for 4094 SNPs. Samples have been color-coded according to the pool name which represents subspecies and country of origin as listed in Table 1.

**Additional file 5: Figure S3.** Histogram of the prediction probabilities for the out-of-sample data. The 90% assignment threshold includes 2098 out of the 2505 samples (=84%).

**Additional file 6: Figure S4.** Confusion matrix for out-of-sample data prediction of evolutionary lineage: African lineage (A), Central and Eastern European lineage (C), Western and Northern European lineage, and Near East and Central Asian lineage (O). Each row of the matrix represents the true class (lineage), while each column represents the predicted class based on the highest prediction probability. The resulting percentages compare a list of expected values with a list of predictions from the model.

**Additional file 7: Figure S5.** Learning curve for the best performing model, the Linear SVC, with average and standard deviation of 10 fold cross validation scores.

**Additional file 8: Table S1.** Additional sampling information, references and sample origin of pool sequencing samples.

**Additional file 9: Table S2.** Pool sequencing and variants statistics.

**Additional file 10: Table S3.** Distribution of selected informative SNPs across the honey bee genome.

**Additional file 11: Table S4.** Average call rate per sample for each subspecies.

**Additional file 12: Table S5.** Accuracy statistics for the different tested models estimated using 10-fold cross validation.

## Data Availability

All sequence data from the pools analyzed during the current study have been submitted to the NCBI Short Read Archive (SRA) under the BioProject accession number PRJNA666033: https://www.ncbi.nlm.nih.gov/sra/?term=PRJNA666033. The pipeline for the analysis of the pool sequence data is available at https://github.com/jlanga/smsk_popoolation.

## Abbreviations

DNA: Deoxyribonucleic acid
FTA: Flinders Technology Associates
ICZN: International Code of Zoological Nomenclature
ML: Machine learning
mtDNA: Mitochondrial deoxyribonucleic acid
PCA: Principal component analysis
PCR: Polymerase chain reaction
SD: Standard deviation
SNP: Single-nucleotide polymorphism
SVC: Support Vector Classifier
SVM: Support Vector Maschine
t-SNE: t-Distributed stochastic neighbor embedding
