# Supplementary material for: Authoritative subspecies diagnosis tool for European honey bees based on ancestry informative SNPs
Source: BMC Genomics. 2021 Feb 3;22:101. doi: 10.1186/s12864-021-07379-7 (PMC7860026; doi:10.1186/s12864-021-07379-7)
Supplement: Supplementary file 2 — Additional file 2: Supplementary materials and methods. Supplementary materials and methods describing in detail the datasets used, the laboratory methods, the bioinformatic pipeline, the SNP selection approach, and the sample classification using Machine Learning algorithms. [file 12864_2021_7379_MOESM2_ESM.pdf]

Supplementary material and methods -

Authoritative subspecies diagnosis tool for European honey bees based on ancestry informative SNPs

Authors: Jamal Momeni[1§*], Melanie Parejo[2,3§], Rasmus O. Nielsen[1], Jorge Langa[2], Iratxe Montes[2], Laetitia Papoutsis[4], Leila Farajzadeh[5], Christian Bendixen[5Δ], SMARTBEES WP3 DIVERSITY COLLABORATORS (in alphabetical order[6-36]) Eliza Căuia[6], Jean-Daniel Charrière[3], Mary F. Coffey[7], Cecilia Costa[8], Raffaele Dall'Olio[9], Pilar De la Rúa[10], M. Maja Drazic[11], Janja Filipi[12], Thomas Galea[13], Miroljub Golubovski[14], Ales Gregorc[15], Karina Grigoryan[16], Fani Hatjina[17], Rustem Ilyasov[18,19], Evgeniya Ivanova[20], Irakli Janashia[21], Irfan Kandemir[22], Aikaterini Karatasou[23], Meral Kekecoglu[24], Nikola Kezic[25], Enikö Sz. Matray[26], David Mifsud[27], Rudolf Moosbeckhofer[28], Alexei G. Nikolenko[19], Alexandros Papachristoforou[29], Plamen Petrov[30], M. Alice Pinto[31], Aleksandr V. Poskryakov[19], Aglyam Y. Sharipov[32], Adrian Siceanu[6], M. Ihsan Soysal[33], Aleksandar Uzunov[34,35], Marion Zammit-Mangion[36], Rikke Vingborg[1#], Maria Bouga[4#], Per Kryger[37#], Marina D. Meixner[34#], Andone Estonba[2#*]

[§]shared first author

[#]equal contributions

[Δ]Deceased

*Corresponding authors: JamalMomeni@eurofins.dk; andone.estonba@ehu.eus

Affiliations:

[1]Eurofins Genomics Europe Genotyping A/S (EFEG), Aarhus, Denmark. (Former GenoSkan A/S, Aarhus, Denmark)
[2]Lab. Genetics, University of the Basque Country (UPV/EHU), Leioa-Bilbao, Spain.
[3]Swiss Bee Research Center, Agroscope, Bern, Switzerland.
[4]Lab of Agricultural Zoology and Entomology, Agricultural University of Athens, Athens, Greece.
[5]Department of Molecular Biology and Genetics, Aarhus University, Aarhus, Denmark.
[6]Institutul de Cercetare Dezvoltare pentru Apicultura SA, Bucharest, Romania
[7]University of Limerick, Ireland
[8]CREA Research Centre for Agriculture and Environment, Italy
[9]BeeSources, Bologna, Italy
[10]Veterinary Faculty, University of Murcia, Spain
[11]Croatian Ministry of Agriculture, Zagreb, Croatia
[12] Department of ecology, agronomy and aquaculture, University of Zadar, Croatia
[13]Breeds of Origin, Malta
[14]MacBee Association, North Macedonia
[15]University of Maribor, Faculty of Agriculture and Life Sciences, Slovenia
[16]Yerevan State University, Armenia
[17]Agricultural Organization 'DEMETER', Dept. of Apiculture,, Greece

[18]Division of Life Sciences, Major of Biological Sciences, and Convergence Research Center for Insect Vectors, Incheon National University, Korea
[19]Institute of Biochemistry and Genetics, Ufa Federal Research Centre of the Russian Academy of Sciences, Russia
[20]University of Plovdiv "Paisii Hilendarski", Bulgaria
[21]Agricultural University of Georgia, Georgia
[22]Ankara University, Turkey
[23]Federation of Greek Beekeepers' Associations, Greece
[24]Düzce University, Turkey
[25]University of Zagreb, Croatia
[26]Hungarian Bee Breeders Association, Budapest, Hungary
[27]Division of Rural Sciences and Food Systems, Institute of Earth Systems, University of Malta, Malta
[28]Österreichische Agentur für Gesundheit und Ernährungssicherheit GmbH, Austria
[29]Cyprus University of Technology, Cyprus
[30]Agricultural University of Plovdiv, Bulgaria
[31]Centro de Investigação de Montanha (CIMO), Instituto Politécnico de Bragança, Portugal
[32]Shulgan-Tash Nature Reserve, Russia
[33]Tekirdag University, Turkey
[34]Landesbetrieb Landwirtschaft Hessen, Bee Institute Kirchhain, Kirchhain, Germany
[35]University Ss. Cyril and Methodius, Faculty of Agricultural Sciences and Food, Skopje, Republic of Macedonia
[36]Department of Physiology and Biochemistry, University of Malta
[37]Department of Agroecology, Aarhus University, Slagelse, Denmark

Supplementary materials and methods

1. Datasets 3
2. Preliminary morphometric analysis 3
3. DNA extraction, library preparation, pool-sequencing 3
4. Bioinformatic processing 3
5. Simulation using allele frequencies from pool sequencing 4
6. SNP selection 4
7. Sample classification using Machine Learning algorithms 5
8. References 6

1. Datasets

Different samples sets have been used for this study:

(1) Whole-genome sequenced pools (total # of individual samples 2145): The pools consisted of around 100 workers bees sampled from across Europe and neighbouring regions (Tables 1 and S1).

(2) Genotypes from pools samples: Most individuals used for pool sequencing were also genotyped individually (N=2050). Of these, 62 outlier samples were excluded after genotyping leaving N=1988. Randomly selected 70% of these samples were used to train the classification model (N=1391), while the remaining 30% were used for validation (N=597).

(3) Newly added samples (N=1908): Additional samples from various subspecies and geographical locations (Table 1) were genotyped for validation and are together with 597 individually genotyped pool samples considered as out-of-sample data (N=2505. These mostly originated from other SmartBees working groups and collaborators.

2. Preliminary morphometric analysis

For some samples included in the pool sequencing, genetic analyses from other studies were available to make sure representative subspecies samples were included in our pools (Table S1). For some of the populations, we conducted preliminary morphometric analyses which were compared to reference data from the Morphometric Bee Data Bank in Oberursel, Germany. Representative subsets from each sampled population were subjected to morphometric analysis, which consisted of dissection of 10 worker bees from each colony under analysis and measurement of 38 morphological characters (in the categories size, pigmentation, pilosity and wing venation) according to methods described in (1) and (2).

3. DNA extraction, library preparation, pool-sequencing

DNA was extracted from the heads or thoraxes of all the worker bees that were homogenized utilizing Qiagen TissueLyser. 50 mg of the homogenized samples were used in DNA purification using MN Blood kit (NucleoMag® Blood 100 µL, Macherey-Nagel, Germany). For each sequencing pool, individual DNAs were mixed in equimolar concentration. Sequencing libraries of the mixed DNA for each pool were constructed with the TruSeq DNA PCR-Free library preparation kit form Illumina. Samples were multiplexed using different tags in order to run on the same flow cell channel. The quality of libraries was assessed by 2100 Bioanalyzer (Agilent Technologies, Santa Clara, CA, USA). Pooled samples were clustered onto flow cells using a Paired-End Cluster Generation Kit HiSeq SBS Kit v4 as per manufacturer protocol (Illumina, San Diego, CA). Sequencing of the pools was carried out using the Illumina HiSeq 2500 platform (V2.2.58) (Eurofins/Genoskan, Denmark). Image analysis was performed using the Illumina pipeline (v.1.8.4).

4. Bioinformatic processing

Two slightly different workflows for the processing and analysis of the raw sequencing data were employed for PCA- and for F_{ST}-based SNP selection:

Data processing for PCA-based SNP selection: Illumina adaptors and low-quality bases were removed using Trimmomatic v0.32 (3) and read quality was checked with FastQC (http://broadinstitute.github.io/fastqc/). High-quality sequences were mapped against the Amel4.5

reference genome (4) using bwa mem 0.7.10 (5). SAMtools v0.1.19 (6) and Picard-tools v1.124 (http://broadinstitute.github.io/picard/) were used to convert between SAM and BAM formats, remove duplicate reads, sort the BAM files, remove reads with low-quality mapping (MAPQ < 20) and retain only properly mapped pairs. Subsequently, the data was processed following the steps of the PoPoolation package (7). The mapping files were split by chromosome, converted to mpileup, and indels were removed. Finally, using a minimum count of 3, the data for the different pools were subsampled to uniform coverage (50x). The entire pipeline is available at https://github.com/jlanga/smsk_popoolation.

Data processing for F_{ST}-based SNP selection: Illumina adaptors and low-quality bases were removed using Cutadapt (8). The trimmed reads were subjected to alignment using BWA-mem (0.7.17) as described in Kofler et al. 2016 to the reference genome of *A. mellifera* (v4.5). Using Picard MarkDuplicates (http://broadinstitute.github.io/picard) and samtools (v0.1.19) (6) the duplicate reads and ambiguous alignments was removed, respectively. For each pool, we retained only high quality alignments in sorted BAM files, which were utilized for variant calling. The detailed mapping statistics were obtained with Samtools and custom Python scripts for each pool. Mpileup files were generated with samtools (v0.1.19) (6) which were introduced as input files to popoolation2 to produce sync files (7) using a minimum quality of 20. Analyses was performed in all major chromosomes (1-16) and 5305 contigs in *A. mellifera* (v4.5) (4). Allele frequency differences and a set of high-quality SNPs were called across all libraries using a minimum sequence quality of 20 and a minimum count of ten.

5. Simulation using allele frequencies from pool sequencing

The pool sequencing approach allows analysis of a larger number of samples per population (~100 individuals per pool) resulting in very accurate estimates of allele frequencies. These were used in order to have a first idea of the population structure using PCA and to have a preliminary evaluation on the performance of the selected SNPs for lineage and subspecies assignment. To this end, we simulated individual genotypes based on allele frequencies of each pool for each selected SNP. With a custom-made R script, we generated 100 diploid genotypes using a random binomial distribution using as probability the allele frequency for each population and SNP. This assumes that the SNPs are independent and in Hardy-Weinberg equilibrium. With the resulting simulated genotypes we performed PCA and plotted the first three components using R. For a higher within-lineage resolution, we also performed PCA and plotted the significant components for each lineage separately. The visual representation already point towards the discriminatory power of the PCA approach (Figure S1). The simulation therefore provided a higher level of confidence to the results.

6. SNP selection

Prior to selection, we dismissed all SNPs with an adjacent SNP or N in the 50 base pair flanking region. This step was necessary to maximize the genotyping success according to Illumina's requirements and left a subset of 370 K SNPs for SNP selection at the first two hierarchical levels:

(1) SNPs for evolutionary lineages: To further reduce the dataset, two additional filtering steps were applied: (i) removing all SNPs closer than 5 kb to each other and, (ii) retaining only SNPs for which the overall minor allele was fixed (MAF=1) in at least one pool. These filters reduce redundant information in the data (see 6,7), leaving a subset of 11 K independent and discriminative SNPs which were then used to perform PCA. The four evolutionary lineages A, M, O and C were clearly differentiated by the first three PCs (Figure S1A). For each SNP the sums of contributions to the first three PCs were calculated and the top 300 SNPs were chosen.

(2) SNPs for subspecies within evolutionary lineages: The data set was split into subspecies belonging to the same evolutionary lineage. As in the previous step, SNPs closer than 5 kb were removed but the MAF filter was loosened to MAF>0.7.

- *M-lineage*: MAF and linkage filters left 5120 SNPs for PCA analysis. There are only two European M-lineage subspecies clearly separated by the first PC. We therefore selected the top 200 SNPs based on their contribution to PC1.
- *O-lineage*: MAF and linkage filters resulted in 4245 SNPs. Our data set contained four subspecies within the O-lineage separated by the first three components. The top 600 SNPs ranked according to the sum of their contributions to the first three PCs were selected.
- *C-lineage*: 2575 SNPs were retained for analysis after MAF and linkage filters. This evolutionary lineage is the most complex with at least 5 subspecies represented in our dataset. Consequently, we selected a higher number of SNPs, namely 1100 SNPs based on the sums of their contributions to the first five PCs.
- *A-lineage*: No SNPs were specifically selected for the African lineage, since only one representative, *A. m. ruttneri* from Malta, is contained in our data set, which is already differentiated in the third PC from the previous step.

(3) SNPs to differentiate populations: To select additional SNPs that can differentiate between pools, pairwise F_{ST} values (11) between all population pairs were calculated for each SNP with two settings (loose and stringent options) using popoolation2 (7). The loose setting option will return more SNPs with less certainty and lower quality, which in turn potentially reduces genotyping success. This drawback is counterbalanced since the loose option increases the chance of identifying highly informative population specific (unique) SNPs. In more detail, the loose option settings comprised a minimum count=4, minimum coverage=5, maximum coverage=250 and a pool size=200. Setting for the stringent option included minimum count=10, minimum coverage=30 and a specific maximum coverage calculated from the average genome coverage for each pool, respectively. The average genome coverage of each pool was calculated using BEDtools (12), and the maximum coverage for each pool was calculated as the mean plus two standard deviations.

Finally, for either setting option (loose and stringent), the pairwise F_{ST} values of each pool against all other pools were summed up for each SNP, and then ranked according to the highest summed F_{ST} value. A fixed and unique SNP in one pool is expected to have a maximum sum of 21, which means this variant is only present in this specific population. A reasonable trade-off between unique and reliable SNPs was achieved by selecting the top 20 SNPs with highest summed F_{ST} from the loose option and the top 80 SNPs from the stringent option for each pool. With 22 pools, a total 2200 informative population-specific SNPs were selected using F_{ST}.

7. Sample classification using Machine Learning algorithms

Machine Learning (ML) algorithms was used to classify and predict assignment of new samples of European subspecies using the selected SNPs. ML algorithms use computational methods to learn information directly from the data without relying on a predetermined equation as a model. The algorithms adaptively improve their performance as the number of samples available for learning increases. In our study candidate SNPs and genotyping data was used to train the model and subsequently the model was used to predict the status of out of data samples.

For this purpose, we used classification machine learning algorithm in Python3 and used Scikit-learn (https://scikit-learn.org/stable/) (13), which is a versatile and open source machine learning framework for Python applications. Five key libraries including scipy, numpy, matplotlib, pandas and

sklearn are installed that are require for initiating the project. In addition, home-built python scripts are prepared for analyzing and visualization of the plots. Then genotyping final report data was loaded and changed with pandas commands to one-hot encoding data because machine learning algorithms require numerical inputs. One-hot encoding is the technique to convert categorical values into a 1-dimensional numerical vector. The resulting vector will have only one element equal to 1 called hot and the rest will be 0 or cold. Each genotype is encoded into three columns, for example, a homozygous reference genotype is encoded as [1,0,0], while a heterozygous variant is encoded as [0,1,0] and a homozygous alternate genotype as [0,0,1]. Missing data is encoded as [0,0,0].

The data of the 1995 samples were split into a training and out-of-sample subset: Data was shuffled, then 70% of the samples (N=1396) were used to train the model and subsequently the model was used to predict the status of out-of-sample data consisting of the remaining 30% of data plus additional samples (N=2507), that were not included in the pooled sequencing. Models were trained and created using the training data. We used different machine learning algorithms to find the best model, among others: Decision Tree Classifier (CART), K Neighbors Classifier (KNN), Linear Discriminant Analysis (LDA), Logistic Regression (LR), Gaussian NB (NB), Random Forest Classifier (RF), Support vector machines (SVM) and linear Support Vector Classification (linear SVC). To estimate the accuracy of the predictive models, 10-fold Cross Validation was applied. Cross Validation is an internal validation technique which involves reserving a specific subset of a training dataset on which the model is not trained. Later, the model is tested this subset before finalizing the model. Table S5 shows the accuracy of the tested models based on Cross Validation. Average accuracy of the models demonstrated that the Linear SVC is the most accurate model. We used this model for further analysis.

The accuracy estimation of the models is easily visualized using Learning Curve (Figure S5). Learning curve refers to a plot of the prediction accuracy versus the training set size that describes the improvement of the model at predicting the target, relative to an increase of the number of instances used to train.

8. References

1. Ruttner F. Biogeography and taxonomy of honeybees. Berlin: Springer Verlag; 1988.

2. Meixner MD, Pinto MA, Bouga M, Kryger P, Ivanova E, Fuchs S. Standard methods for characterising subspecies and ecotypes of *Apis mellifera*. J Apic Res. 2013;52.

3. Bolger, A. M., Lohse, M. & Usadel, B. Trimmomatic: a flexible trimmer for Illumina sequence data. Bioinformatics **30**, 2114–2120 (2014).

4. Elsik CG, Worley KC, Bennett AK, Beye M, Camara F, Childers CP, et al. Finding the missing honey bee genes: Lessons learned from a genome upgrade. BMC Genomics. 2014;15.

5. Li H, Durbin R. Fast and accurate short read alignment with Burrows-Wheeler transform. Bioinformatics. 2009;25:1754–1760.

6. Li H, Handsaker B, Wysoker A, Fennell T, Ruan J, Homer N, et al. The Sequence Alignment/Map format and SAMtools. Bioinformatics. 2009;25:2078–2079.

7. Kofler R, Pandey RV, Schlötterer C. PoPoolation2: identifying differentiation between populations using sequencing of pooled DNA samples (Pool-Seq. Bioinformatics. 2011;27(24):3435–3436.

8. Martin M. Cutadapt removes adapter sequences from high-throughput sequencing reads. EMBnet.journal. 2011 May;S.l.], v. 17, n. 1:10–12,.

9. Paschou P, Drineas P, Lewis J, Nievergelt CM, Nickerson DA, Smith JD, et al. Tracing Sub-Structure in the European American Population with PCA-Informative Markers. PLoS Genet. 2008;4:1000114.

10. Lewis J, Abas Z, Dadousis C, Lykidis D, Paschou P, Drineas P. Tracing cattle breeds with principal components analysis ancestry informative SNPs. PloS one. 2011;6(4).

11. Weir BS, Cockerham CC. Estimating F-statistics for the analysis of population structure. Evolution. 1984;38:1358–1370.

12. Quinlan AR, Hall IM. BEDTools: a flexible suite of utilities for comparing genomic features. Bioinformatics. 2010;26(6):841–842.

13. Pedregosa F, Varoquaux G, Gramfort A, Michel V, Thirion B, Grisel O, et al. Scikit-learn: Machine learning in Python. Journal of machine learning research. 2011;12(Oct):2825–2830.
