# Supplementary figures and images for: Authoritative subspecies diagnosis tool for European honey bees based on ancestry informative SNPs
Source: BMC Genomics. 2021 Feb 3;22:101. doi: 10.1186/s12864-021-07379-7 (PMC7860026; doi:10.1186/s12864-021-07379-7)

## A) Evolutionary lineages

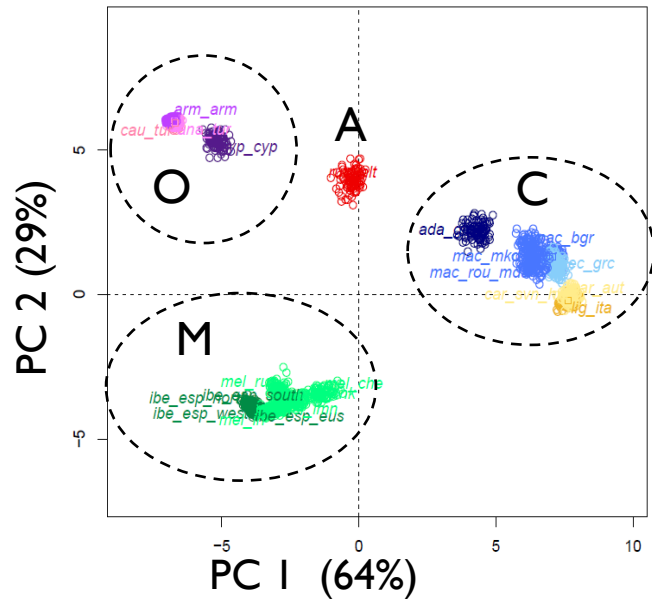

B) M-lineage ssp.

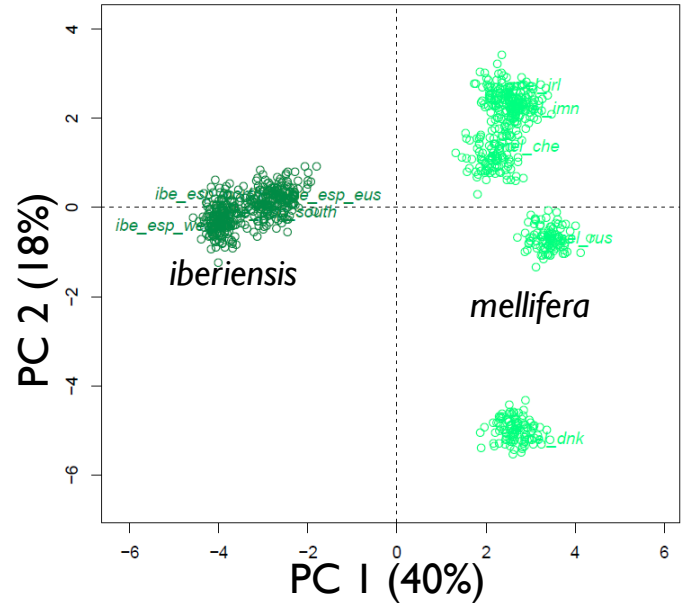

C) O-lineage ssp.

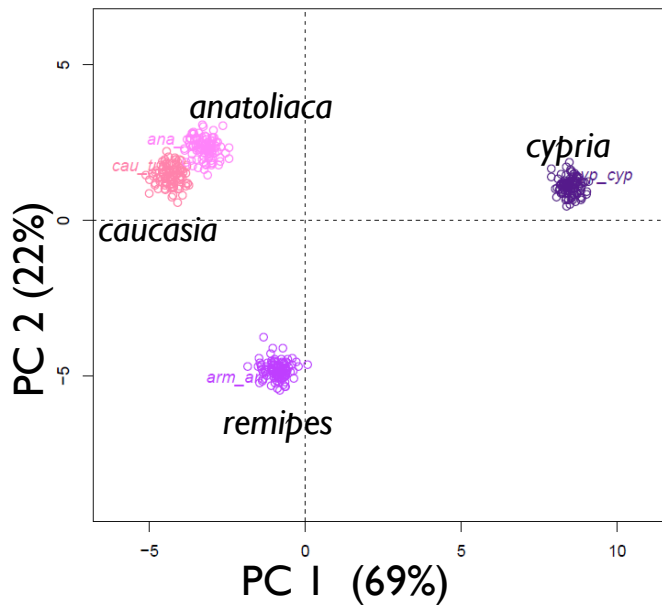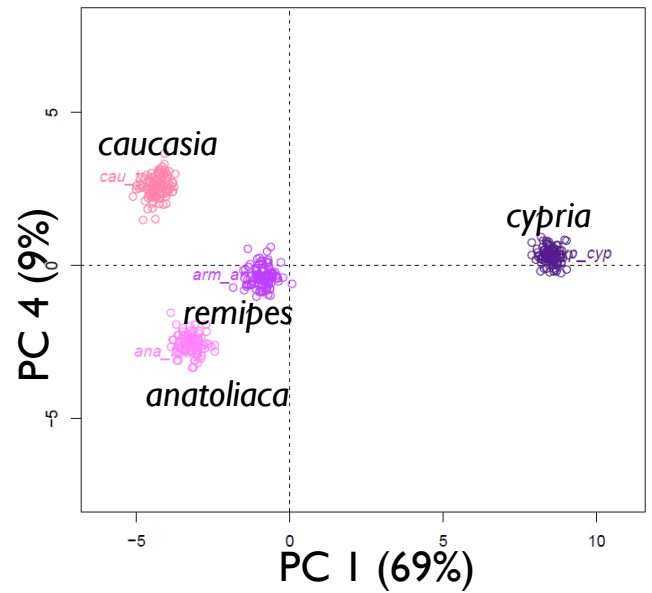

D) C-lineage ssp.

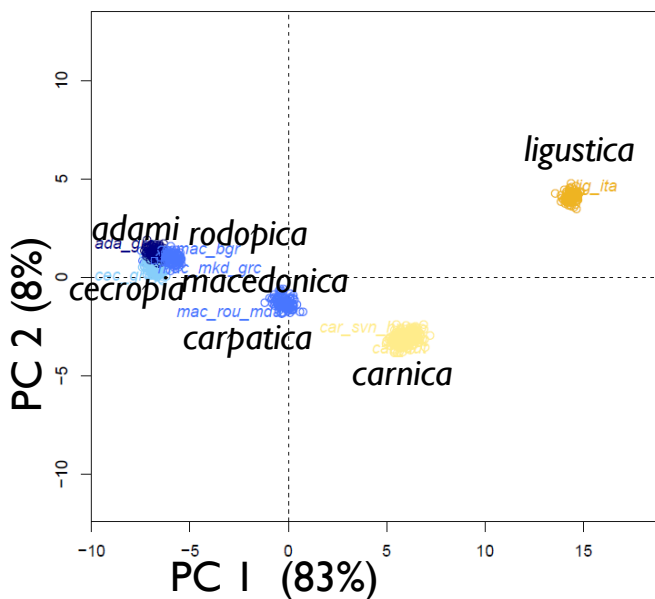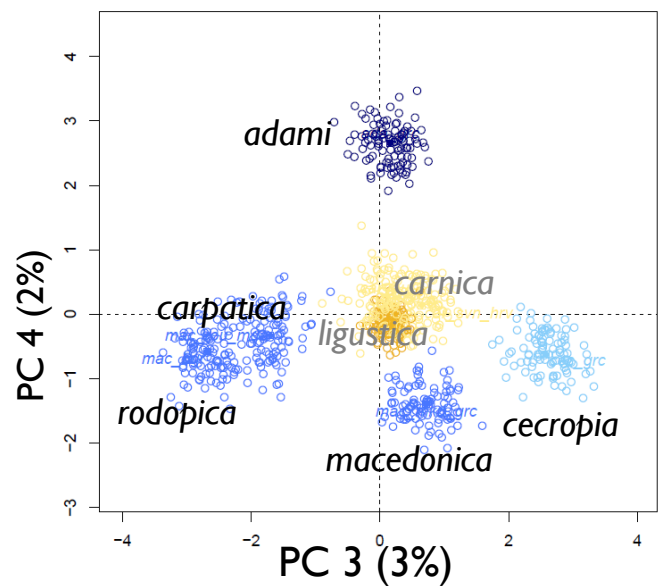

Supplement: Supplementary file 3 — Additional file 3: Figure S1. PCA-Plots with the PCA-selected SNPs and 100 simulated individuals based on allele frequencies of the pools. (A) Using 300 SNPs, the evolutionary lineages M, C and O were well separated with the first two PCs, while lineage A can be differentiated with the third component (not shown). (B) Within the M-lineage, harboring only two European subspecies (A. m. mellifera and A. m. iberiensis), the first PC using 200 SNPs already contributes sufficient information. (C) In the O-lineage, four subspecies are represented that are separated with 600 SNPs using 4 PCs. (D) For lineage C that contains numerous subspecies (subspecies complex), 1100 SNPs were selected to obtain a better resolution. [file 12864_2021_7379_MOESM3_ESM.pdf]

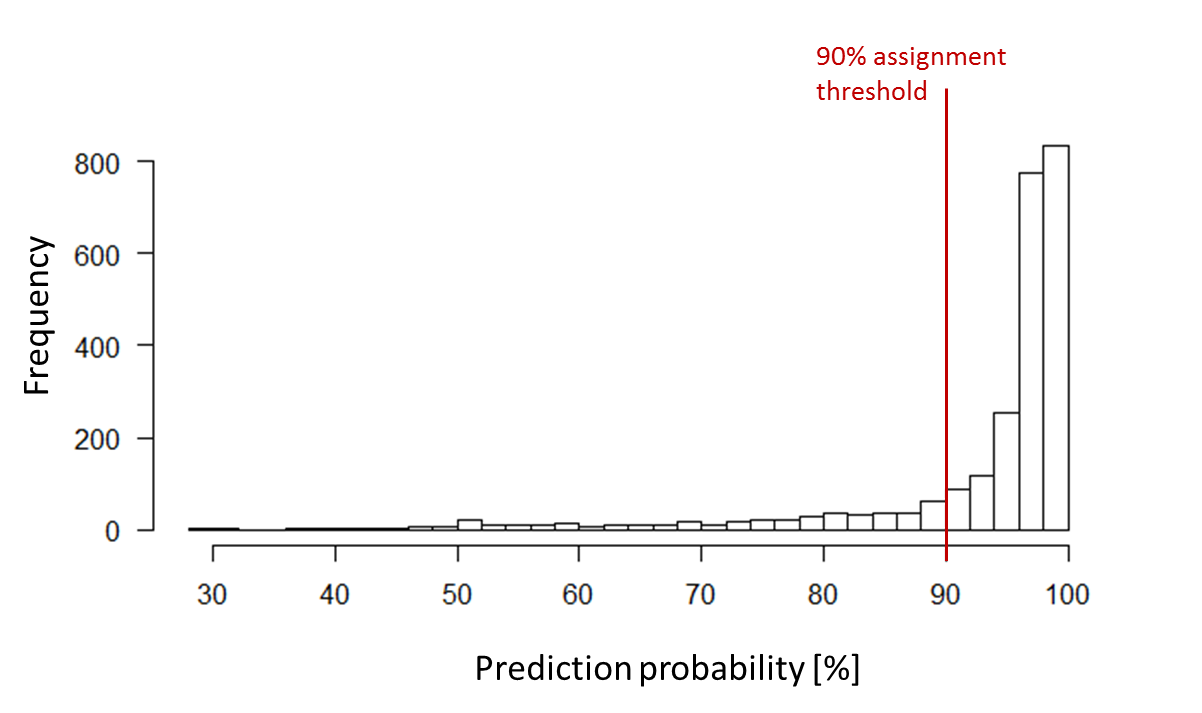

Supplement: Supplementary file 5 — Additional file 5: Figure S3. Histogram of the prediction probabilities for the out-of-sample data. The 90% assignment threshold includes 2098 out of the 2505 samples (=84%). [file 12864_2021_7379_MOESM5_ESM.png]

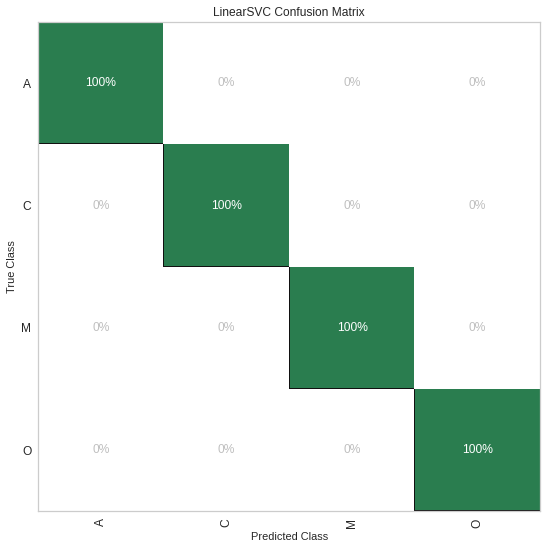

Supplement: Supplementary file 6 — Additional file 6: Figure S4. Confusion matrix for out-of-sample data prediction of evolutionary lineage: African lineage (A), Central and Eastern European lineage (C), Western and Northern European lineage, and Near East and Central Asian lineage (O). Each row of the matrix represents the true class (lineage), while each column represents the predicted class based on the highest prediction probability. The resulting percentages compare a list of expected values with a list of predictions from the model. [file 12864_2021_7379_MOESM6_ESM.png]

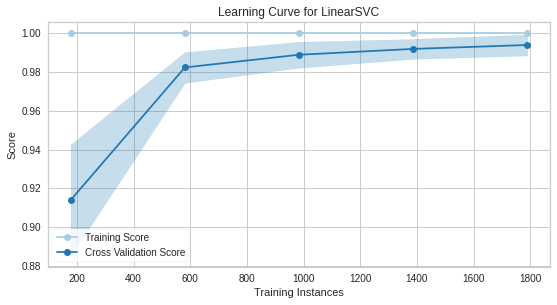

Supplement: Supplementary file 7 — Additional file 7: Figure S5. Learning curve for the best performing model, the Linear SVC, with average and standard deviation of 10 fold cross validation scores. [file 12864_2021_7379_MOESM7_ESM.png]
