# Supplementary material for: Authoritative subspecies diagnosis tool for European honey bees based on ancestry informative SNPs
Source: BMC Genomics. 2021 Feb 3;22:101. doi: 10.1186/s12864-021-07379-7 (PMC7860026; doi:10.1186/s12864-021-07379-7)
Supplement: Supplementary file 4 — Additional file 4: Figure S2. Visualization using a t-SNE manifold plot of the 1988 honey bee samples from the pool sequencing individually genotyped for 4094 SNPs. Samples have been color-coded according to the pool name which represents subspecies and country of origin as listed in Table 1. [file 12864_2021_7379_MOESM4_ESM.pdf]

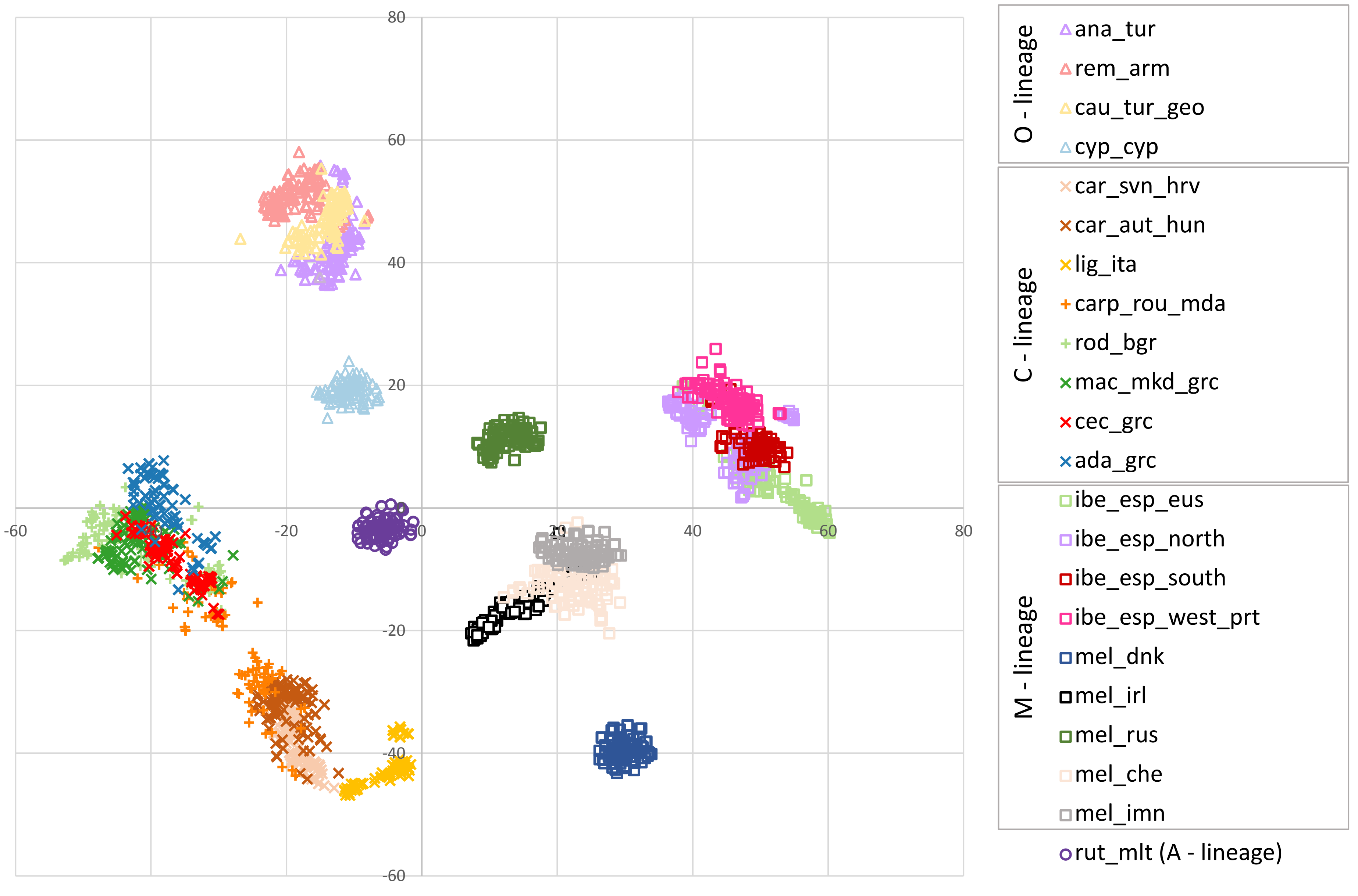
80
60
40
20
-20
-40
-60
0
20
40
60
80
O - lineage
ana_tur
rem_arm
cau_tur_geo
cyp_cyp
C - lineage
car_svn_hrv
car_aut_hun
lig_ita
carp_rou_mda
rod_bgr
mac_mkd_grc
cec_grc
ada_grc
M - lineage
ibe_esp_eus
ibe_esp_north
ibe_esp_south
ibe_esp_west_prt
mel_dnk
mel_irl
mel_rus
mel_che
mel_imn
rut_mlt (A - lineage)
